# iCircDA-NEAE: Accelerated attribute network embedding and dynamic convolutional autoencoder for circRNA-disease associations prediction

**DOI:** 10.1371/journal.pcbi.1011344

**Published:** 2023-08-31

**Authors:** Lin Yuan, Jiawang Zhao, Zhen Shen, Qinhu Zhang, Yushui Geng, Chun-Hou Zheng, De-Shuang Huang

**Affiliations:** 1 Key Laboratory of Computing Power Network and Information Security, Ministry of Education, Shandong Computer Science Center, Qilu University of Technology (Shandong Academy of Sciences), Jinan, China; 2 Shandong Engineering Research Center of Big Data Applied Technology, Faculty of Computer Science and Technology, Qilu University of Technology (Shandong Academy of Sciences), Jinan, China; 3 Shandong Provincial Key Laboratory of Computer Networks, Shandong Fundamental Research Center for Computer Science, Jinan, China; 4 School of Computer and Software, Nanyang Institute of Technology, Nanyang, China; 5 Eastern Institute for Advanced Study, Eastern Institute of Technology, Ningbo, China; 6 Key Lab of Intelligent Computing and Signal Processing of Ministry of Education, School of Artificial Intelligence, Anhui University, Hefei, China; Peking University, CHINA

## Abstract

Accumulating evidence suggests that circRNAs play crucial roles in human diseases. CircRNA-disease association prediction is extremely helpful in understanding pathogenesis, diagnosis, and prevention, as well as identifying relevant biomarkers. During the past few years, a large number of deep learning (DL) based methods have been proposed for predicting circRNA-disease association and achieved impressive prediction performance. However, there are two main drawbacks to these methods. The first is these methods underutilize biometric information in the data. Second, the features extracted by these methods are not outstanding to represent association characteristics between circRNAs and diseases. In this study, we developed a novel deep learning model, named iCircDA-NEAE, to predict circRNA-disease associations. In particular, we use disease semantic similarity, Gaussian interaction profile kernel, circRNA expression profile similarity, and Jaccard similarity simultaneously for the first time, and extract hidden features based on accelerated attribute network embedding (AANE) and dynamic convolutional autoencoder (DCAE). Experimental results on the circR2Disease dataset show that iCircDA-NEAE outperforms other competing methods significantly. Besides, 16 of the top 20 circRNA-disease pairs with the highest prediction scores were validated by relevant literature. Furthermore, we observe that iCircDA-NEAE can effectively predict new potential circRNA-disease associations.

## Author summary

CircRNA-disease association prediction is extremely helpful in understanding pathogenesis, diagnosis, and prevention, as well as identifying relevant biomarkers. In this paper, we proposed a novel deep learning-based method called iCircDA-NEAE to discover new potential circRNA-disease associations. Experimental results demonstrated that iCircDA-NEAE outperforms other state-of-the-art prediction methods, and can accurately predict potential circRNA-disease associations. Furthermore, according to the relevant literature, we observed that novel circRNA-disease associations predicted by iCircDA-NEAE are potential associations. The performance of iCircDA-NEAE mainly depends on three factors: (i) iCircDA-NEAE incorporates multi-source biometric information to measure complex associations between circRNAs and diseases. (ii) iCircDA-NEAE uses disease semantic similarity, Gaussian interaction kernel (GIP), circRNA expression profile similarity, and Jaccard similarity to make the most of biometric information in the data. (iii) iCircDA-NEAE incorporates the advantages of ANNE and DCAE, which not only effectively integrates multi-source information, but also effectively captures hidden high-level information of data.

## Introduction

Circular RNAs (circRNAs) are a class of non-coding RNA characterized by a covalently closed-loop structure generated through a special type of alternative splicing termed back-splicing. Given that circRNAs lack free ends and are thus relatively stable, they are abundant in the eukaryotic transcriptomes. It has been shown that circRNAs are involved in various life activities of organisms, including functioning as microRNA (miRNA) sponges [1], regulating alternative splicing [2], modulating the expression of parental genes [3], etc. In addition, accumulating evidence suggests that circRNAs affect many diseases, such as glioma [4], breast cancer [5], and liver cancer [6]. Therefore, the study of circRNAs is crucial for disease diagnosis and treatment.

At present, identifying circRNA-disease associations is appealing to find potential biomarkers and understand the diagnosis and treatment of diseases. However, the circRNA-disease associations are very complicated and remain still obscure. With the development of sequencing and analysis technology, various biological experiments have emerged to identify circRNA-disease associations [7–9]. However, biological experiments are generally costly and labor-intensive. The experimentally supported circRNA-disease association databases (circ2Disease [10], circRNADisease [11], circR2Disease [12], circ2Traits [13], circFunbase [14]) provide an opportunity to develop computational methods for circRNA-disease association identification.

Recently, researchers have proposed many deep learning-based methods to predict circRNA-disease associations. For example, GCNCDA [15], one of the most well-verified DL-based algorithms, applied graph convolutional network to predict circRNA-disease associations. ASAECDA [16], another impressive DL-based algorithm, calculated weight values of the links between circRNAs and diseases based on graph embedding and stacked autoencoder. GATCDA [17] used graph attention network to predict scores for unknown circRNA-disease associations. IMS-CDA [18] identified potential circRNA-disease associations by incorporating multi-source similarity information into a deep stacked autoencoder model. iCDA-CGR [19] used chaos game representation technology to discover the associations between circRNAs and diseases. RNMFLP predicted circRNA-disease associations based on robust nonnegative matrix factorization and label propagation [20]. iGRLCDA identified circRNA-disease association based on graph representation learning [21]. These methods achieved impressive prediction performance. However, we found that these methods suffer from two major drawbacks. The first is these methods underutilize biometric information in the data. Second, the features used by these methods are not outstanding to represent association characteristics between circRNAs and diseases.

**Fig 1:**
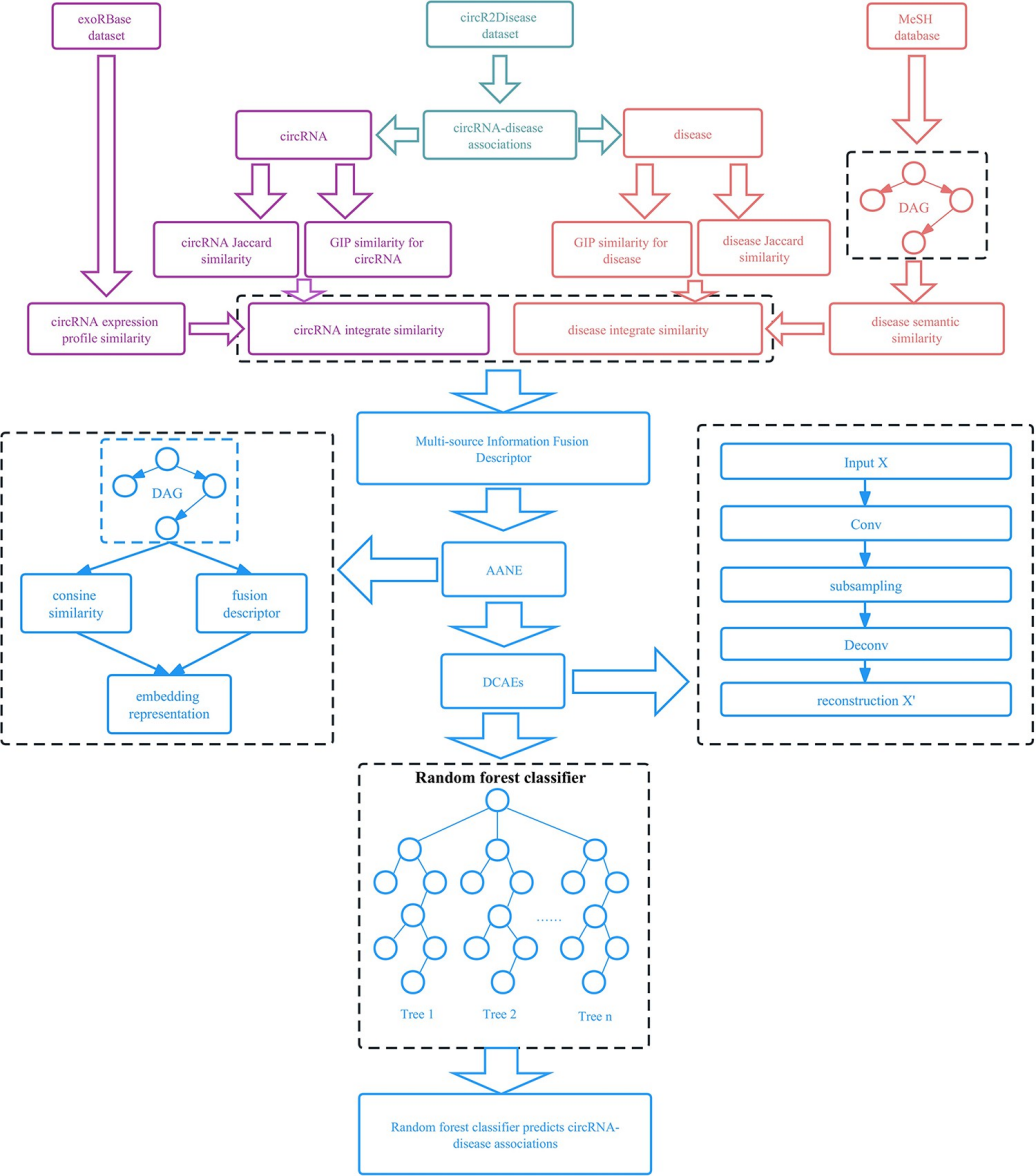
Schematic overview of iCircDA-NEAE framework. Experimental data comes from exoRBase dataset, circR2Disease dataset and MeSH dataset. Disease semantic similarity, Gaussian interaction kernel (GIP), circRNA expression profile similarity, and Jaccard similarity are used to measure the biometric information in the data, then multisource information fusion descriptor was constructed. AANE and DCAE are used to learn the features in the data. Random forest classifier are used to predict circRNA-disease association.

In this study, we developed a novel deep learning model for identifying Circrna-Disease Associations based on accelerated attribute Network Embedding and dynamic convolutional AutoEncoder (iCircDA-NEAE). The proposed model iCircDA-NEAE can (i) make the most of the bio-metric information in the data (ii) enhance the feature extraction capability of the model by using multiple feature extraction methods, and (iii) predict circRNA-disease associations accurately. Specifically, (i) circRNA-disease association data were collected from the circR2Disease database; (ii) disease semantic similarity, Gaussian interaction kernel (GIP), circRNA expression profile similarity, and Jaccard similarity were used to measure the biometric information in the data, then multisource information fusion descriptor was constructed; (iii) accelerated attribute network embedding (AANE) extracts features from the descriptor data; (IV) dynamic convolutional autoencoder (DCAE) extracts hidden features from data; (V) random forest classifier used hidden features to predict circRNA-disease association. The schematic overview of iCircDA-NEAE framework is shown in Fig 1. 5-fold and 10-fold cross-validation on training data and test data experiments were used to validate the model performance. Experimental results show that iCircDA-NEAE outperforms other competing methods significantly. Furthermore, according to the relevant literature, we observe that novel circRNA-disease associations predicted by iCircDA-NEAE are potential associations.

## Results

### Hyperparameter Selection of iCircDA-NEAE

In a random forest classifier, max_feature determines the number of features in each decision tree. Too small max_feature may contain incomplete feature information, while too large max_feature led to overfitting problems. In this section, the important hyperparameter max_feature was investigated experimentally, whereas other hyperparameters were set to default values.

**Fig 2:**
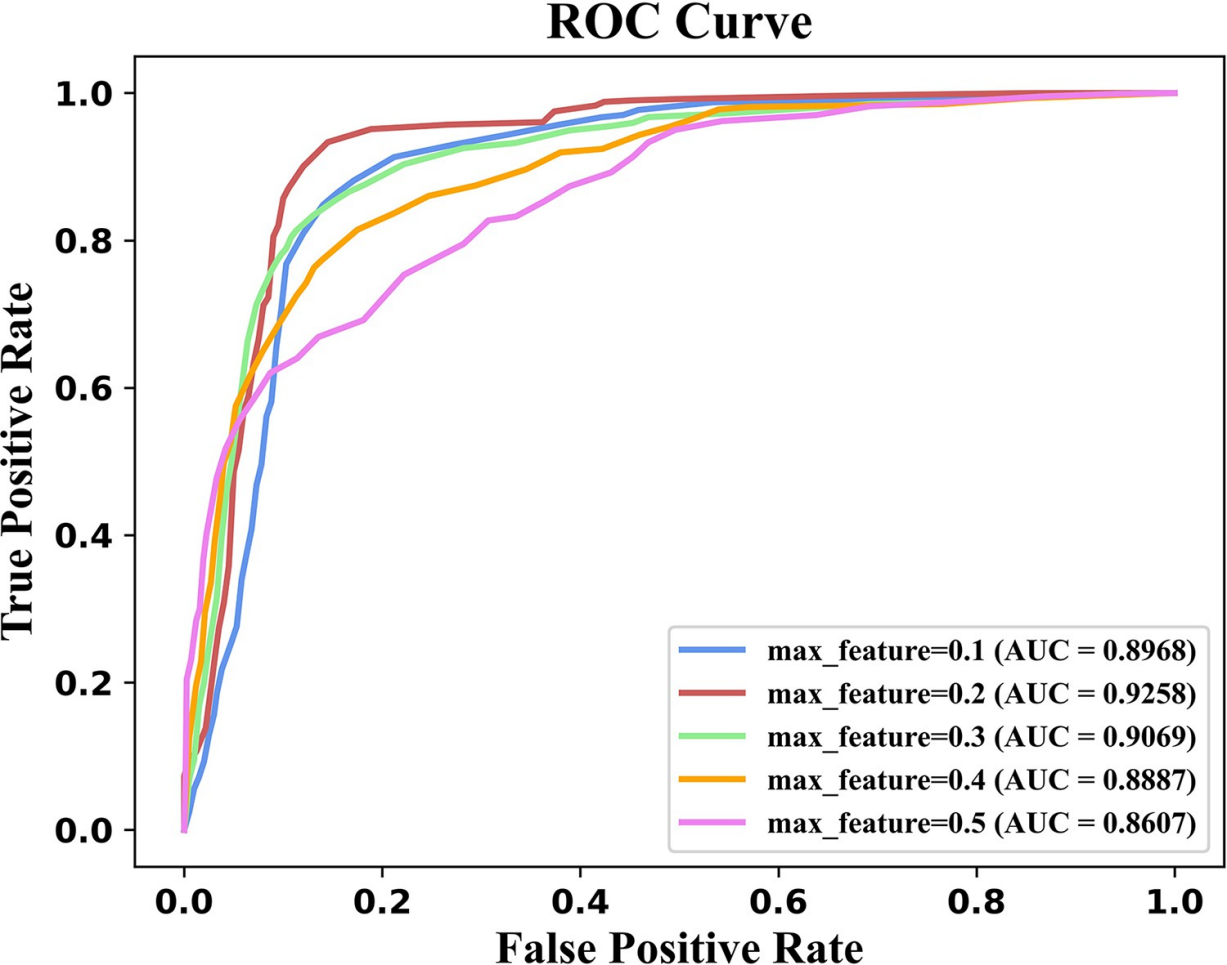
Comparison of model performance under different max_feature values. AUC value of iCircDA-NEAE is the highest when max_feature is set to 0.2.

The value of max_feature ranges from 0.1 to 0.5 [22]. As shown in Fig 2, the AUC value of iCircDA-NEAE is the highest when max_feature is set to 0.2. Therefore, in this experiment, we set max_feature to 0.2.

### Contribution of AANE and DCAE

In this section, the effects of AANE and DCAE were evaluated by ablation experiments with five different models. Specifically, (i) iCircDA-NEAE without AANE; (ii) iCircDA-NEAE without DCAE; (iii) iCircDA-NEAE without AANE and DCAE; (IV) DCAE replaced by CAE in iCircDA-NEAE; (V) AANE replaced by NE in iCircDA-NEAE.

As shown in Table 1, when we remove AANE or DCAE, the performance drops by about 8%, and after removing both two feature extraction models, the model suffers significant performance degradation. Furthermore, after replacing DCAE and AANE with CAE and NE respectively, both models give worse results than our proposed iCircDA-NEAE model. Experimental results show that both AANE and DCAE are beneficial to circRNA-disease association prediction, and the model outperforms traditional network embedding and convolutional autoencoder.

**Table 1:** Ablation study of iCircDA-NEAE with different kinds of feature extraction model.

Methods	circR2Disease	circRNAdisease	circ2Disease
iCircDA-NEAE	**0.9258**	**0.9203**	**0.9214**
(w/o) AANE	0.8512	0.8437	0.8520
(w/o) DCAE	0.8506	0.8413	0.8510
(w/o) DCAE & AANE	0.8015	0.7986	0.8114
AANE & CAE	0.8802	0.8806	0.8812
NE & DCAE	0.8841	0.8814	0.8770

Note: w/o means without. AANE and DCAE represent accelerated attribute network embedding and dynamic convolutional autoencoder, respectively. CAE and NE represent convolutional autoencoder and network embedding, respectively.

We compared the run time of iCircDA-NEAE with iCircDA-NEAE’ (DCAE replaced by CAE) on the NVIDIA RTX 3080 GPU with 10GB of VRAM. Experimental results show that the computation time (63 min 27 s) of iCircDA-NEAE is less than that (80 min 23 s) of iCircDA-NEAE’. CAE model are computationally more expensive than DCAE model. The detailed results were recorded in S1 Table.

### Comparison with different classifiers

In this section, we compared iCircDA-NEAE with traditional machine learning algorithms as well as common deep learning algorithms, including SVM (Support Vector Machine) [23], RF (Rotation Forest) classifier [24], DNN (Deep Neural Network) [25] and XGBoost [26]. To make the results comparable, we only replaced the classifier in the model with the classifier that need to be compared. The detailed parameters of all classifiers were presented in Table 2.

**Table 2:** The detailed parameters of all classifiers.

Method	Parameter	
RF	Max_feature = 0.2, n_estimators = 100	
SVM	Probability = True, kernel = ‘poly’	
DNN	Three fully connected layers, Use ReLU and sigmoid as activation functions of hidden layers and output layer	Optimizer = Adadelta Loss = mean-squared error
Rotation forest	set the number of feature subsets as 4, the number of decision trees as 3	
XGBoost	penalty parameter = 0.1, max_depth = 8	

**Fig 3:**
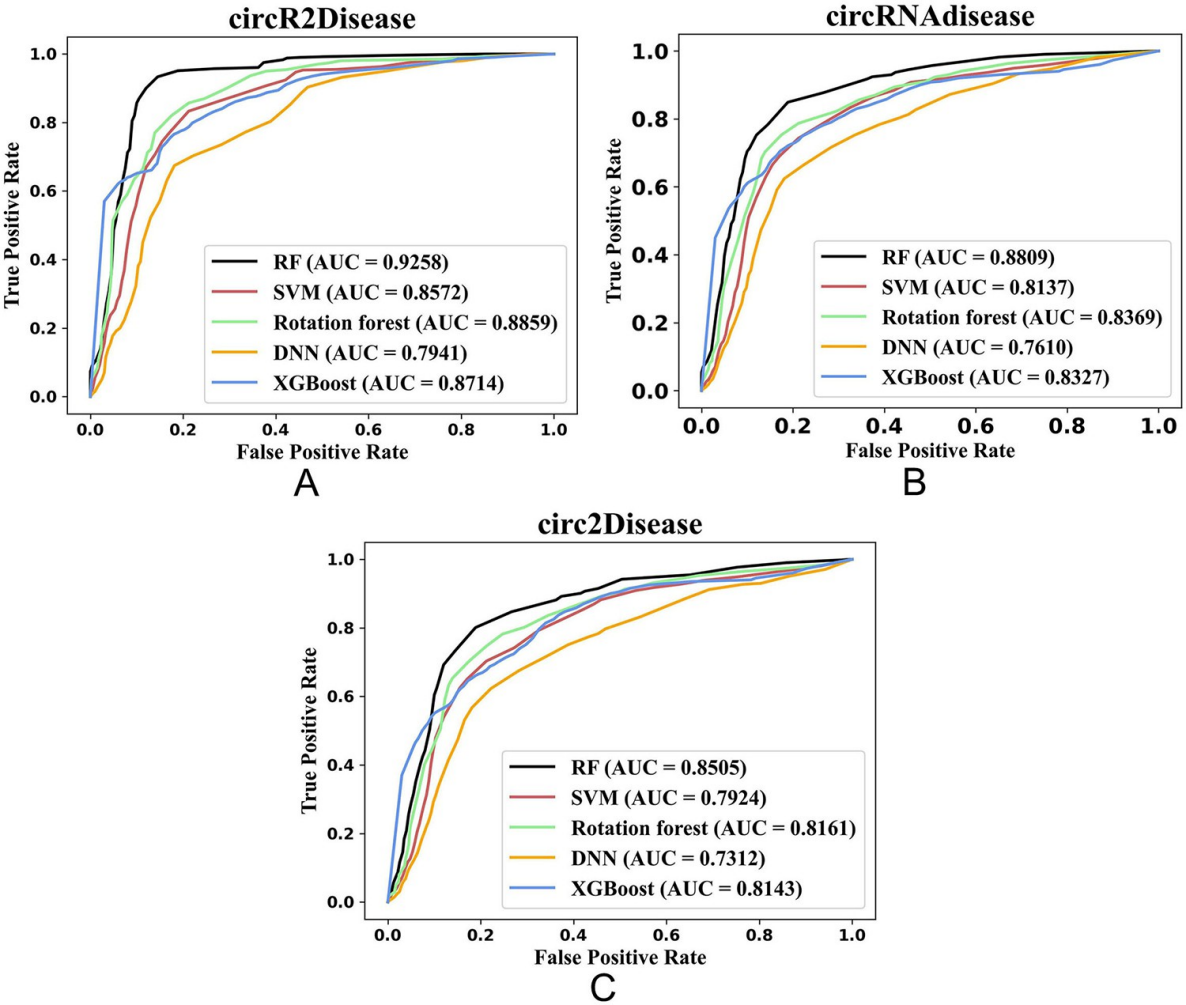
The performance of iCircDA-NEAE with five classifiers on three datasets. (A) The performance on circR2Disease dataset. (B) The performance on circRNAdisease dataset. (C) The performance on circ2Disease dataset.

We compared the performance of iCircDA-NEAE with the five classifiers by using benchmark dataset and two independent datasets (circR-NAdisease and circ2Disease datasets). The ROC curves on the three datasets were shown in Fig 3A–3C, respectively. As shown in Fig 3, iCircDA-NEAE with random forest classifier outperforms other classifiers on all datasets. The ACC, Sen, F1, MCC and AUC values were presented in Table 3. As shown in Table 3, iCircDA-NEAE with random forest classifier outperforms other classifiers on all evaluation metrics.

**Table 3:** The performance of iCircDA-NEAE with the five classifiers by using benchmark dataset and two independent datasets (circRNAdisease and circ2Disease datasets).

Datasets	Classifier	ACC	Sen	F1	MCC	AUC
circR2Disease	RF	**0.9273**	**0.9165**	**0.8939**	**0.8261**	**0.9258**
	SVM	0.8631	0.8023	0.8456	0.8077	0.8572
	DNN	0.8004	0.8051	0.7992	0.7594	0.7941
	Rotation forest	0.8876	0.8579	0.8475	0.7942	0.8859
	XGBoost	0.8768	0.8257	0.8341	0.7832	0.8714
circRNAdisease	RF	**0.8682**	**0.8355**	**0.8327**	**0.7113**	**0.8809**
	SVM	0.8105	0.7730	0.7508	0.6118	0.8137
	DNN	0.7893	0.7584	0.7316	0.6305	0.7610
	Rotation forest	0.8217	0.8098	0.7884	0.6260	0.8369
	XGBoost	0.8422	0.8034	0.8045	0.7011	0.8327
circ2Disease	RF	**0.8487**	**0.7325**	**0.7170**	**0.4327**	**0.8505**
	SVM	0.7951	0.6961	0.6482	0.3241	0.7924
	DNN	0.7580	0.6706	0.6513	0.3586	0.7312
	Rotation forest	0.8128	0.7148	0.6879	0.4039	0.8161
	XGBoost	0.8090	0.7037	0.6719	0.4276	0.8143

### Comparison of different datasets

**Fig 4:**
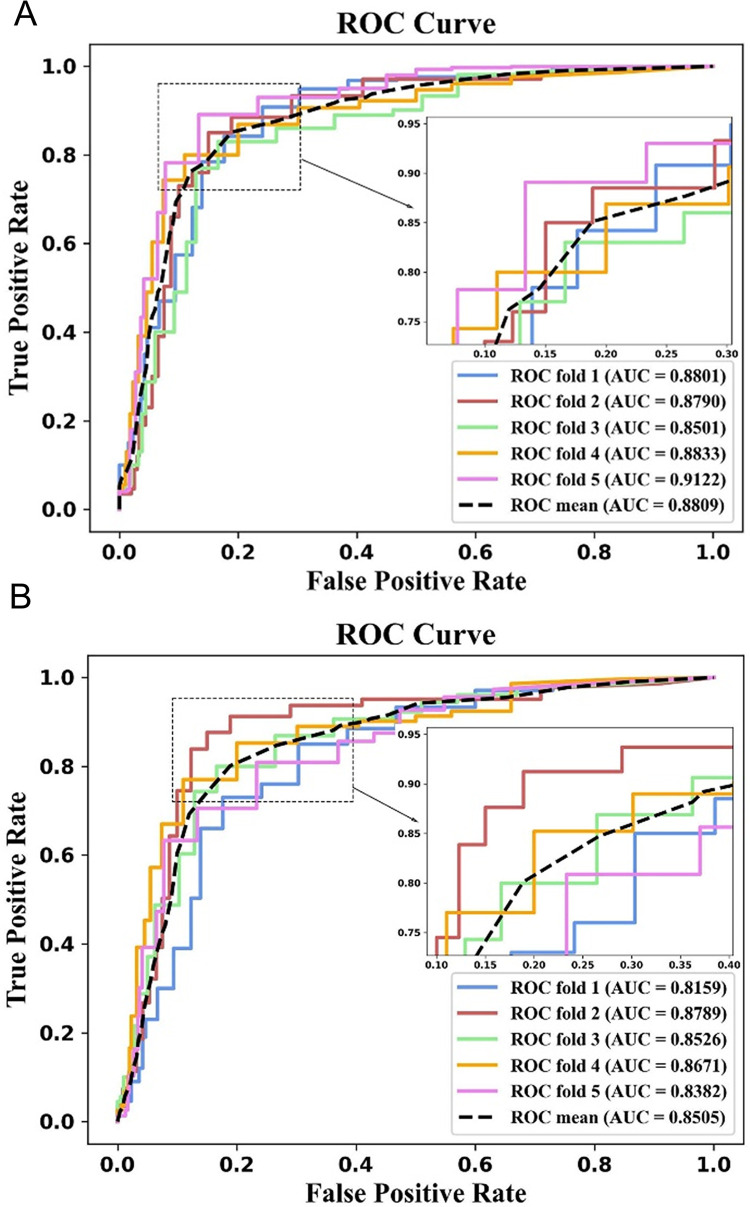
The performance of iCircDA-NEAE on circRNAdisease and circ2Disease datasets. (A) AUC values of iCircDA-NEAE on the circRNAdisease dataset. (B) AUC values of iCircDA-NEAE on the circ2Disease dataset.

In this section, the model performance was evaluated by using two independent datasets (circRNAdisease dataset and circ2Disease dataset) with 5-fold and 10-fold cross-validation. As shown in Fig 4, the AUC values of iCircDA-NEAE on the circRNAdisease and circ2Disease datasets are 0.8809 and 0.8505 respectively. The 5-fold cross-validation experimental results on the circRNAdisease and circ2Disease datasets were presented in Table 4. For the circRNAdisease dataset, the ACC, Sen, F1 and MCC of iCircDA-NEAE are 0.8682, 0.8335, 0.8327 and 0.6613, respectively. For the circ2Disease dataset, the ACC, Sen, F1 and MCC of iCircDA-NEAE are 0.8487, 0.7325, 0.7170 and 0.4327, respectively. The 10-fold cross-validation experimental results were presented in S2 and S3 Tables, respectively. For circRNAdisease dataset, the ACC, Sen, F1, MCC and AUC of iCircDA-NEAE are 0.8735, 0.8413, 0.8274, 0.6635 and 0.8962, respectively. For the circ2Disease dataset, the ACC, Sen, F1, MCC and AUC of iCircDA-NEAE are 0.8537, 0.7530, 0.7074, 0.4341 and 0.8575, respectively. These results suggest that iCircDA-NEAE can achieve good prediction performance on several important datasets.

**Table 4:** The 5-fold cross-validation experimental results on the circRNAdisease and circ2Disease datasets.

Datasets	Test set	ACC	Sen	F1	MCC	AUC
circRNAdisease	1-fold	0.8347	0.8260	0.8512	0.6329	0.8801
	2-fold	0.8561	0.8469	0.8200	0.6512	0.8790
	3-fold	0.9014	0.8577	0.8377	0.7044	0.8501
	4-fold	0.8675	0.8356	0.8325	0.6917	0.8833
	5-fold	0.8819	0.8113	0.8221	0.6263	0.9122
	Average	**0.8682**	**0.8355**	**0.8327**	**0.6613**	**0.8809**
circ2Disease	1-fold	0.8976	0.7259	0.6912	0.4826	0.8159
	2-fold	0.8618	0.7435	0.7254	0.4518	0.8789
	3-fold	0.9022	0.7580	0.7291	0.4075	0.8526
	4-fold	0.8173	0.7341	0.7133	0.4226	0.8671
	5-fold	0.7646	0.7012	0.7260	0.3990	0.8382
	Average	**0.8487**	**0.7325**	**0.7170**	**0.4327**	**0.8505**

### Comparison with other methods

**Fig 5:**
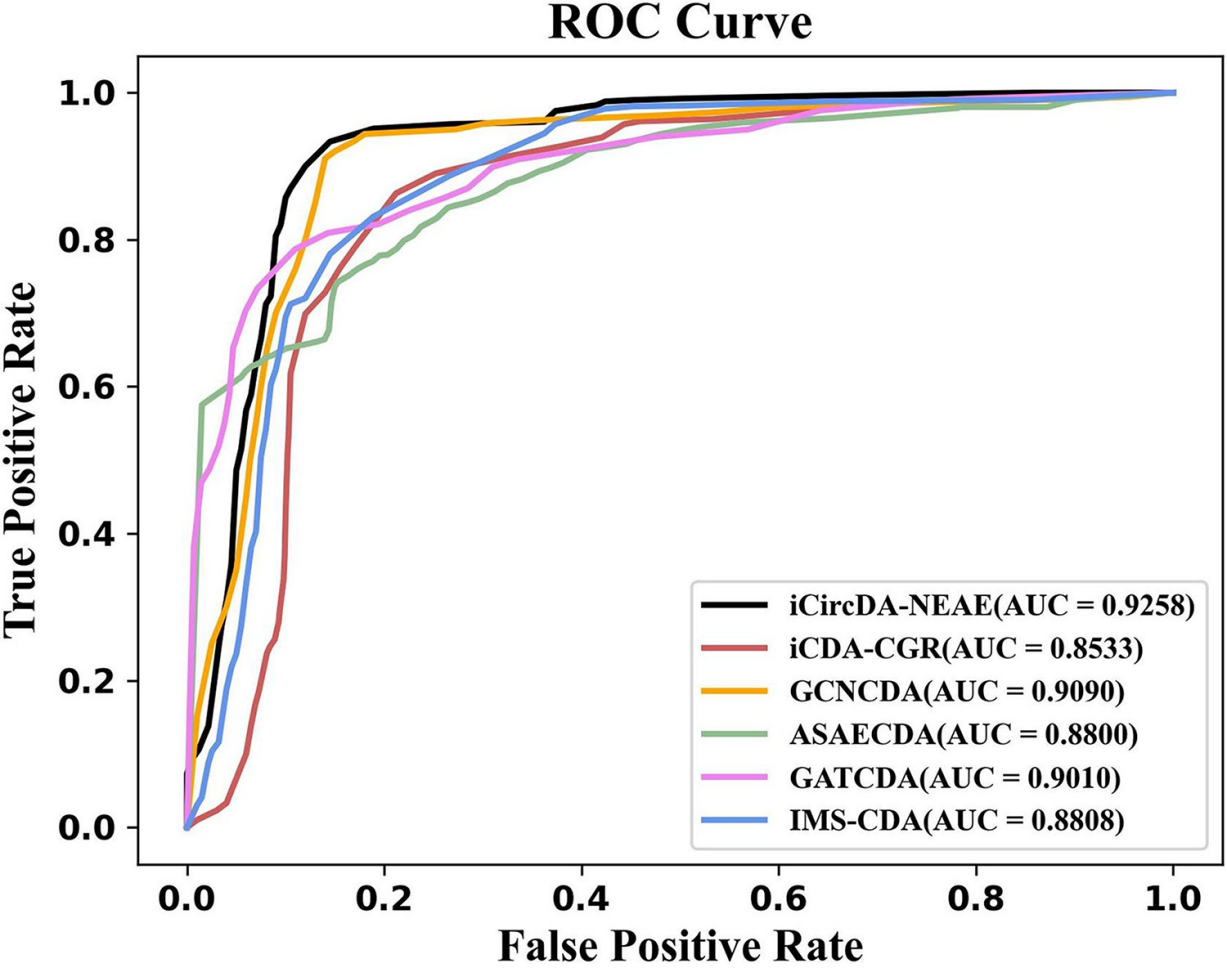
Performance comparison of iCircDA-NEAE and the competing methods on the benchmark dataset.

In this section, we used 5-fold cross-validation to compare the performance of iCircDA-NEAE with five state-of-the-art circRNA-disease association prediction models, including iCDA-CGR [19], GCNCDA [15], ASAECDA [16], GATCDA [17] and IMS-CDA [18]. All models were run on a widely used benchmark dataset circR2Disease. As shown in Fig 5, iCircDA-NEAE outperforms other state-of-the-art prediction methods significantly.

In terms of features, although these state-of-the-art methods have used a variety of feature information, they can consider more biometric information. Our proposed iCircDA-NEAE considers both circRNA expression profile similarity and Jaccard similarity. To the best of our knowledge, we are the first to use both circRNA expression profile similarity and Jaccard similarity to predict circRNA-disease associations. Furthermore, our method performs multi-source feature fusion, which can measure the correlation of multiple feature information and fuse this information into a unified information identifier. At the same time, features without redundant information can effectively improve model performance.

In terms of models, these state-of-the-art methods used traditional deep learning or machine learning algorithms. iCDA-CGR used chaos game representation (CGR) technology to quantify the nonlinear relationship of circRNA sequences. However, the model did not deal with redundant information resulting in poor predictive performance. IMS-CDA and ASAECDA are two deep learning methods based on stacked autoencoder (SAE), which use SAE to extract features from multi-source information. Compared with SAE, our proposed DCAE can capture high-level representations of the data. GCNCDA is a GCN (Graph Convolutional Networks)-based prediction method, and GATCDA is a GTN (Graph Attention Network)-based prediction method. Compared with these two methods, iCircDA-NEAE incorporates the advantages of ANNE and DCAE, which not only effectively integrates multi-source information, but also effectively capture hidden high-level information of data.

### Case studies

In this section, we applied iCircDA-NEAE to the benchmark dataset circR2Disease for predicting novel potential circRNA-disease associations. We sorted all unconfirmed circRNA-disease associations in descending order based on their prediction scores. The higher the score, the greater the likelihood of a circRNA-disease association. We selected the top 20 circRNA-disease associations (as shown in Table 5), 17 of which have been confirmed by different databases and literature. For example, hsa_circ_0004214 is highly upregulated in breast cancer and promotes tumorigenesis [27]; hsa_circ_0001785 acts as a diagnostic biomarker in breast cancer treatment [28]; and hsa_circ_0004277 is considered as a potential diagnostic marker and therapeutic target for acute myeloid leukemia [29]. The three unconfirmed circRNA-disease associations are hsa_circ_0046701-lung cancer, hsa_circ_0037911-pancreatic cancer, and hsa_circ_0005836-colorectal cancer. hsa_circ_0046701 promotes carcinogenesis by increasing the expression of ITGB8 in glioma [30], and the expression level of ITGB8 has significantly upregulated in lung cancer tissues compared with normal tissues [31]. These pieces of evidence suggest that hsa_circ_0046701 may serve as a potential biomarker in lung cancer. miRNA-637 suppresses tumorigenesis in pancreatic ductal adenocarcinoma cells [32]. In essential hypertension, has-circ-0037911 was found to suppress miR-637 activity by acting as a sponge [33]. These results show that has-circ-0037911 may promote pancreatic ductal adenocarcinoma by inhibiting miR-637 activity. In pulmonary tuberculosis, hsa_circ_0005836 is related to the regulation of the mTOR signaling pathway [34]. The mTOR signaling pathway is a target for colorectal cancer therapy [35]. These studies suggest that hsa_circ_0005836 may be related to colorectal cancer.

**Table 5:** The top 20 circRNA-disease associations.

Rank	circRNA	Disease	Evidence
1	hsa_circ_0005105	Osteoarthritis	PMID:28276108
2	hsa_circ_0007385	Lung cancer	PMID:29372377
3	hsa_circ_0004214	Glioma	PMID:28622299
4	hsa_circ_0000523	Colorectal cancer	PMID:25624062
5	hsa_circ_0000936	Glioma	circFunbase
6	hsa_circ_0001785	Breast cancer	PMID:29045858
7	hsa_circ_0108942	Breast cancer	circRNAdisease
8	hsa_circ_0007534	Colorectal cancer	PMID:29364478
9	hsa_circ_0001946	Glioma	PMID:26683098
10	hsa_circ_0046701	Lung cancer	unconfirmed
11	hsa_circ_0007534	Breast cancer	PMID:29593432
12	hsa_circ_0037911	Pancreatic ductal adenocarcinoma	unconfirmed
13	hsa_circ_0000504	Colorectal cancer	circRNAdisease
14	hsa_circ_0072088	Liver cancer	PMID:28727484
15	hsa_circ_0000284	Pancreatic cancer	PMID:29255366
16	hsa_circ_0054633	Diabetes mellitus	PMID:27878383
17	hsa_circ_0005836	Colorectal cancer	unconfirmed
18	hsa_circ_0092276	Breast cancer	circRNAdisease
19	hsa_circ_0001649	Colorectal cancer	PMID:29421663
20	hsa_circ_0001187	Acute myeloid leukemia	PMID:28282919

## Discussion

Accumulating evidence suggests that circRNAs play crucial roles in human diseases. CircRNA-disease association prediction is extremely helpful in understanding pathogenesis, diagnosis, and prevention, as well as identifying relevant biomarkers. Therefore, there is an urgent need to develop novel computational methods to accurately predict circRNA-disease associations.

In this paper, we proposed a novel deep learning-based method called iCircDA-NEAE to discover new potential circRNA-disease associations. Experimental results demonstrated that iCircDA-NEAE outperforms other state-of-the-art prediction methods, and can accurately predict potential circRNA-disease associations. Besides, 16 of the top 20 circRNA-disease pairs with the highest prediction scores were validated by relevant literature. Furthermore, according to the relevant literature, we observed that novel circRNA-disease associations predicted by iCircDA-NEAE are potential associations.

The performance of iCircDA-NEAE mainly depends on three factors: (i) iCircDA-NEAE incorporates multi-source biometric information to measure complex associations between circRNAs and diseases. (ii) iCircDA-NEAE uses disease semantic similarity, Gaussian interaction kernel (GIP), circRNA expression profile similarity, and Jaccard similarity to make the most of biometric information in the data. (iii) iCircDA-NEAE incorporates the advantages of ANNE and DCAE, which not only effectively integrates multi-source information, but also effectively captures hidden high-level information of data.

Two possible issues in this paper should be discussed: (i) since negative samples are difficult to obtain, we can only randomly select samples from unconfirmed samples as negative samples. The number of positive samples and negative samples is the same, thus avoiding the sample imbalance problem. But doing this will inevitably lead to negative samples containing very few true positive samples. (ii) since iCircDA-NEAE utilizes the strongly-supervised label information (true association labels) to predict circRNA-disease associations, so iCircDA-NEAE is overwhelmingly dependent on the quality of the ground truth association labels. Therefore, some more comprehensive methods should be proposed to solve the two issues in future works.

## Materials and methods

### Datasets and model

Since circR2Disease (http://bioinfo.snnu.edu.cn/) is the most comprehensive and commonly used database, this study used circR2Disease as the benchmark database. The circRNA expression profiles and disease information were collected from the exoRBase database (http://www.exoRBase.org) [36] and the MeSH database (http://www.nlm.nih.gov/mesh) [37], respectively.

We constructed a sample-balanced circRNA-disease association dataset using the circR2Disease dataset. The association dataset contains 661 circRNAs, 100 diseases, 739 circRNA-disease positive associations, and 739 circRNA-disease negative associations. 739 circRNA-disease positive associations are experimentally validated associations, and 739 circRNA-disease negative associations are randomly selected from 66100 unknown associations of the circR2Disease dataset. The circRNAdisese database contains 330 circRNAs, 48 diseases and 354 circRNA-disease associations. The circ2Disease database contains 249 circRNAs, 61 diseases and 273 circRNA-disease associations.

First, iCircDA-NEAE uses disease semantic similarity, Gaussian interaction kernel (GIP), circRNA expression profile similarity and Jaccard similarity to measure the biometric information in the data, and constructs multisource information fusion descriptor. Second, AANE extracts feature from the descriptor data. Third, DCAE extracts hidden features from data. Finally, the random forest classifier uses hidden features to predict circRNA-disease association. The flow chart of iCircDA-NEAE is shown in Fig 1. The source code and data are available at: https://github.com/nathanyl/iCircDA-NEAE.

### Similarity measures

Before introducing the method, we summarize the notation used in this paper as follows: italic indicates a scalar quantity, as in *A* or *a*; lower case boldface indicates a vector quantity, as in **a**; upper case boldface indicates a matrix quantity, as in **A**.

Similarity measurement can convert the relationship between biological factors into feature information that can be used by the model, so it is a crucial step in building a prediction model. We constructed similarity matrices from four aspects: disease semantic similarity, Gaussian interaction profile kernel, circRNA expression profile similarity, and Jaccard similarity.

### Construction of disease semantic similarity

Disease semantic similarity measures the relationship between diseases [38–40]. The MeSH database uses a directed cycle graph (DAG) to represent diseases and disease associations. A node in the DAG represents a disease, and the edges of the DAG represent associations between diseases. In MeSH, DAG_*d*_(*d*, *N*_*d*_, *E*_*d*_) is used to represent information about disease *d*, N_*d*_ represents the set of disease nodes that are related to *d* and contain *d* itself, and E_*d*_ represents the set of edges between these diseases. For disease *e*, if Nd contains *e* and *e* = *d*, the disease contribution value of *e* to *d* is defined as 1(D_*d*_(*e*) = 1). If *e*≠*d*, the disease contribution value is calculated as follows:

$$\{\begin{array}{ll} D_{d}(e)=1 & \mathrm{if}e=d\\ D_{d}(e)=\max\left\{\mu \cdot D_{d}\left(e^{\prime }\right)e^{\prime }\in \mathrm{children}\;\mathrm{of}\;e\right\} & \mathrm{if}\;e\neq d \end{array}$$ (1)

where *μ* is the semantic contribution factor between diseases, we set *μ* to 0.5 according to the study [41].

Then, the semantic value *DV*(*d*) of disease *d* is defined as follows:

$$DV(d)=\sum_{e\in N_{d}}D_{d}(e)$$ (2)

In DAG, the more nodes are shared between two diseases, the more similar the two diseases are. The semantic similarity **DSS**_**1**_(*d*(*i*), *d*(*j*)) between disease *d*(*i*) and *d*(*j*) is defined as follows:

$$DSS_{1}(d(i),d(j))=\frac{\sum_{e\in N_{d(i)}\cap N_{d(j)}}\left(D_{d(i)}(e)+D_{d(j)}(e)\right)}{DV(d(i))+DV(d(j))}$$ (3)

where **DSS**_**1**_ is the disease semantic similarity matrix.

While considering the disease semantic similarity **DSS**_**1**_, the impact of disease number on disease contribution should also be considered. Inspired by Wang’s method [42], the contribution of disease *e* under the influence of the disease number can be defined as follows:

$$D_{d}^{\prime }(e)=-\log\left(\frac{num\left(DAG_{d}(e)\right)}{num(diseases)}\right)$$ (4)

where *num(DAG*_*d*_*(e))* is the number of diseases associated with disease *d* and *num(diseases)* is the number of all diseases.

Then, the disease semantic similarity **DSS**_**2**_(*d*(*i*), *d*(*j*)) of disease *d*(*i*) and *d*(*j*) can be defined as follows:

$$DSS_{2}(d(i),d(j))=\frac{\sum_{e\in N_{d(i)}\cap N_{d(j)}}\left(D_{d(i)}^{\prime }(e)+D_{d(j)}^{\prime }(e)\right)}{DV(d(i))+DV(d(j))}$$ (5)

### Construction of the Gaussian interaction profile kernel

To obtain comprehensive disease similarity information, we used Gaussian interaction profile (GIP) [43–45] kernel to calculate disease similarity. Assuming that circRNA *c*_*1*_ is associated with disease *d*_*1*_, if disease *d*_*2*_ is highly similar to disease *d*_*1*_, then disease *d*_*2*_-associated circRNAs tend to have similar functions to circRNA *c*_*1*_ [46]. Therefore, we used circRNA-disease association adjacency matrix to calculate the GIP kernel similarity between disease *d*_*i*_ and *d*_*j*_, the formula is defined as follows:

$$GD(d(i),d(j))=\exp\left(-\mu {\left\|V(d(i))-V(d(j))\right\|}^{2}\right)$$ (6)

where GD is the GIP kernel similarity matrix between diseases. *d*(*i*) represents the row vector of the *i*-th disease and *μ* is the bandwidth parameter of the GIP, which can be calculated by the following formula:

$$\mu =\frac{1}{n}\sum_{i=1}^{n}{\left\|V(d(i))\right\|}^{2}$$ (7)

where *n* is the number of rows of the circRNA-disease association matrix.

Similarly, the GIP kernel similarity between circRNAs is defined as follows:

$$GC(c(i),c(j))=\exp\left(-\mu {\left\|V(c(i))-V(c(j))\right\|}^{2}\right)$$ (8)

where GC is the GIP kernel similarity matrix between circRNAs. *c*(*i*) represents the column vector of the *i*-th circRNA and *μ* is the bandwidth parameter of the GIP, which can be calculated by the following formula:

$$\mu =\frac{1}{m}\sum_{i=1}^{m}{\left\|V(c(i))\right\|}^{2}$$ (9)

where *m* is the number of columns of the circRNA-disease association matrix.

### Construction of the CircRNA expression profile similarity

The circRNA expression profile (EP) similarity from exoRBase data-base is another important information for constructing circRNA-disease association prediction models. We used 32-dimensional feature vectors to represent circRNAs, and sorted the circRNAs in descending order according to the feature vectors [16,47,48]. Spearman correlation coefficient [49] was used to calculate the EP similarity between circRNAs:

$$\rho \left(c_{i},c_{j}\right)=1-\frac{6\sum d_{p}^{2}}{k(k-1)},\;d_{p}=l_{p}^{i}-l_{p}^{j}$$ (10)

where *d*_*p*_ is the feature vector difference between circRNA *i* and circRNA *j*, *l*^*i*^ represents the 32-dimensional vector of *i*-th circRNA after sorting, and *k* is the number of circRNAs. Let **SE** be an *k*×*k* circRNA adjacency matrix consisting of *ρ*(*c*_*i*_, *c*_*j*_).

### Construction of the Jaccard similarity

Jaccard similarity is used to represent the similarity between sets [50–52]. *J*(*A*, *B*) is the ratio of the intersection of sets *A* and *B* to the union of *A* and *B*. The larger the Jaccard value, the higher the similarity between sets *A* and *B*. We used Jaccard to calculate the similarities between diseases and circRNAs. We calculated the Jaccard similarity of disease *d*(*i*) and disease *d*(*j*) with the following formula:

$$JD(d(i),d(j))=\left|\frac{ca(d(i))\cap ca(d(j))}{ca(d(i))\cup ca(d(j))}\right|$$ (11)

where **JD** is the Jaccard similarity matrix between diseases. **ca**(*d*(*i*)) represents the circRNAs associated with disease *d*(*i*).

The Jaccard similarity calculation formula of circRNAs is defined as follows:

$$JC(c(i),c(j))=\left|\frac{da(c(i))\cap da(c(j))}{da(c(i))\cup da(c(j))}\right|$$ (12)

where **JC** is the Jaccard similarity matrix between circRNAs. **da**(*c*(*i*)) represents the diseases associated with circRNA *c*(*i*).

### Multisource feature fusion

The multisource feature fusion method can fuse a variety of biological feature information, eliminate redundant information, and improve the accuracy of feature extraction. Feature fusion was used to integrate multiple similarity information into a unified identifier, which contains a large number of circRNA and disease feature information, and contains multiple association information. The fusion of disease similarity multisource in-formation can be defined as follows:

$$DS(d(i),d(j))=\{\begin{array}{ll} \frac{DSS_{1}(d(i),d(j))+DSS_{2}(d(i),d(j))}{2}, & \mathrm{if}\;d(i)\;\mathrm{and}\;d(j)\\ & \mathrm{has}\;\mathrm{semantic}\;\mathrm{similarity}\\ GD(d(i),d(j)), & \mathrm{otherwise} \end{array}$$ (13)

DM=[DS,JD] (14)

The fusion of circRNA similarity multisource information can be defined as follows:

$$CS(c(i),c(j))=\{\begin{array}{ll} \frac{\rho (c(i),c(j))+GC(c(i),c(j))}{2}, & \mathrm{if}\;\rho (c(i),c(j))\;\mathrm{exits}\\ GC(c(i),c(j)), & \mathrm{otherwise} \end{array}$$ (15)

CM=[CS,JC] (16)

Finally, we used principal component analysis (PCA) [53] to reduce the dimensionality of **CM** and **DM**, and obtain **CM** and **DM**. The fusion information of circRNA and disease is obtained according to the following formula:

FV(c(i),d(j))=[CM(c(i)),DM(d(j))] (17)

Among them, **CM**(*c*(*i*)) represents the *i*-th row vector of **CM**, and **DM**(*d*(*j*)) represents the *j*-th column vector of **DM**.

Let **AM** be an *m*×*n* adjacency matrix corresponding to the circRNA-disease association dataset from circR2Disease database, where *m* (*m* = 661) is the number of circRNAs and *n* (*n* = 100) is the number of diseases. If **AM**(*i*, *j*) = 1, it means that circRNA *c*(*i*) is associated with disease d(j), otherwise **AM**(*i*, *j*) = 0.

### Feature extraction methods

#### AANE algorithm to extract features

**Fig 6:**
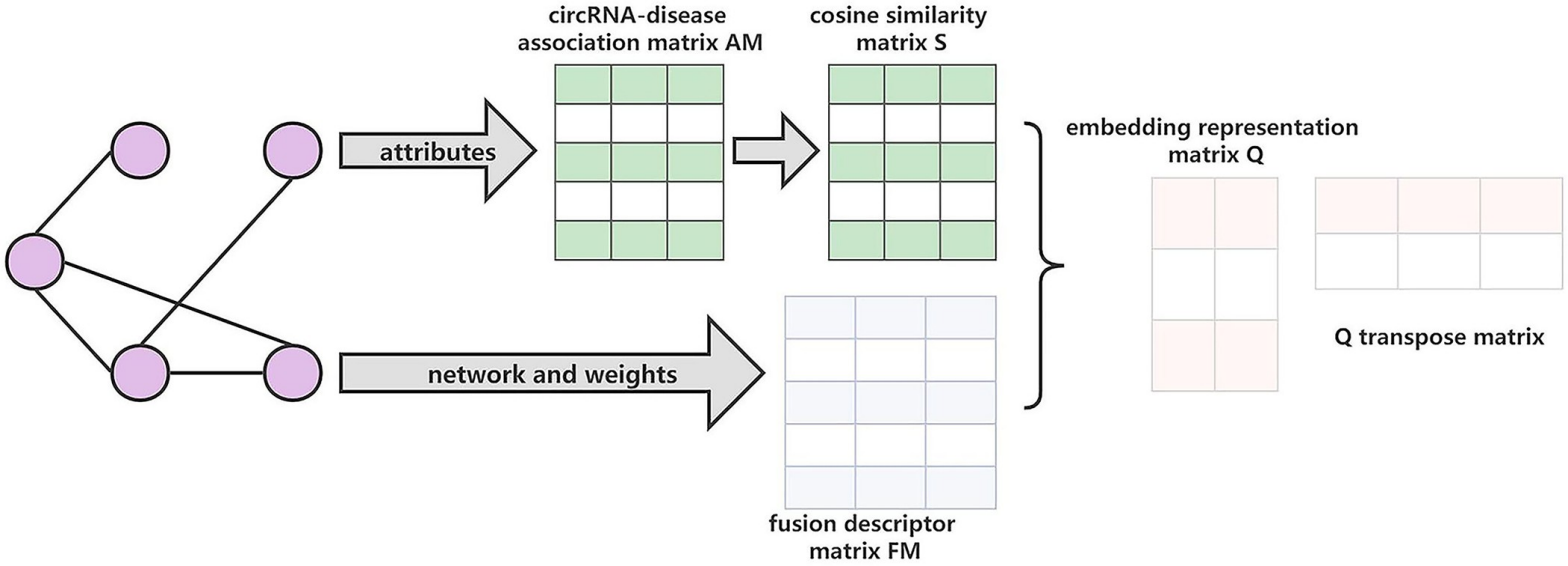
Schematic overview of AANE framework.

Compared with widely used feature extraction methods PCA, LINE (Large-scale Information Network Embedding) [54], node2vec [55] and DeepWalk [56], AANE incorporates the correlation between node attrib-utes into the network embedding to better learn feature representations. AANE is used to extract low-dimensional features. The flowchart of AANE algorithm is shown in Fig 6.

For a network N = (*V*, *E*, *W*), *V* is the node set, *W* is the edge set, and the edge *e*_*ij*_ in *W* represents the edge connecting node *i* and node *j*. The value of *e*_*ij*_ is closely related to the similarity between nodes. The larger the value of *e*_*ij*_, the more similar node *i* is to node *j*. According to the theory that a real symmetric matrix can be diagonalized by an orthogonal matrix, the formula is defined as follows:

$$A=H\Lambda H^{T}=HB^{2}H^{T}=HBH^{T}HBH^{T}=\left(HBH^{T}\right){\left(HBH^{T}\right)}^{T}=UU^{T}$$ (18)

where **A** is a semi-definite symmetric matrix, which can be represented by an orthogonal matrix **H** and a diagonal matrix **Λ**. **B** is a matrix consisting of the square root of the elements in the **Λ**.

When applying this algorithm, the similarity matrix **S** is calculated by applying the cosine similarity algorithm to the attribute matrix **AM**. Based on Eq 18, matrix **S** is decomposed into two matrices **Q** and **Q**^**T**^.


$$S=QQ^{T}$$ (19)

Node vectors have high similarity in two situations, one is that the nodes have high similarity in topological structure, and the other is that the weight value between nodes is large. The objective function is defined as follows:

$$L={\left\|S-QQ^{T}\right\|}_{F}^{2}+\lambda \sum \omega_{ij}{\left\|q_{i}-q_{j}\right\|}^{2}$$ (20)

where *λ* is the balance parameter. Based on **Z** = **Q**, the objective function can be written as follws:

$$L=\sum {\left\|s_{i}-q_{i}Z\right\|}_{2}^{2}+\lambda \sum \omega_{ij}{\left\|q_{i}-z_{i}\right\|}^{2}+\frac{\rho }{2}\sum \left({\left\|q_{i}-z_{i}+u_{i}\right\|}_{2}^{2}-{\left\|u_{i}\right\|}_{2}^{2}\right)$$ (21)

where *q* represents the penalty parameter, and *u*_*i*_ is the scaled data of the dual variable. The alternating direction method of the multiplier (ADMM) is used to solve the objective function:

$$q_{i}^{t+1}=(2s_{i}Z^{t}+\lambda \sum \frac{\omega_{ij}z_{j}^{t}}{\|q_{i}^{t}-z_{j}^{t}{\|}_{2}})+\rho (z_{i}^{t}-u_{i}^{t})P^{-1}P=2{\left(Z^{t}\right)}^{T}Z^{t}+(\lambda \frac{\omega_{ij}}{\|q_{i}^{t}-z_{j}^{t}{\|}_{2}+\rho })I$$ (22)

$$z_{i}^{t+1}=(2s_{i}Q^{t+1}+\lambda \sum \frac{\omega_{ij}q_{j}^{t+1}}{\|z_{i}^{t}-q_{j}^{t+1}{\|}_{2}}+\rho (q_{i}^{t+1}+u_{i}^{t}))L^{-1}L=2{\left(Q^{t+1}\right)}^{T}Q^{t+1}+(\lambda \sum \frac{\omega_{ij}q_{j}^{t+1}}{\|z_{i}^{t}-q_{j}^{t+1}{\|}_{2}}+\rho )I$$ (23)

#### Dynamic convolutional autoencoder to extract features

**Fig 7:**
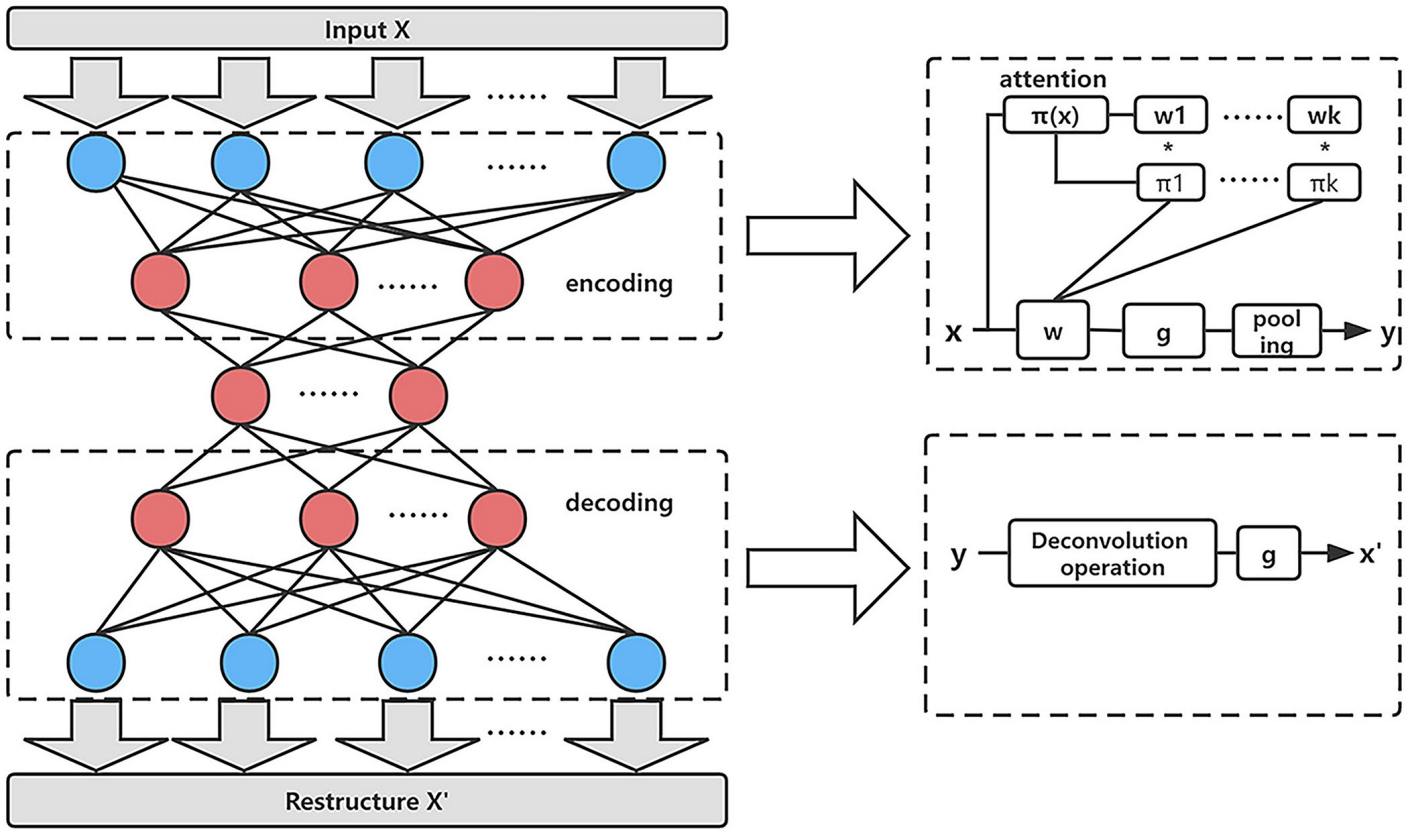
Schematic overview of DCAE framework.

Convolutional autoencoder (CAE) can efficiently extract hidden features from data [57,58]. Inspired by the dynamic convolution [59,60], we proposed a dynamic convolutional autoencoder (DCAE) by replacing the convolution with dynamic convolution. DCAE extracts features more efficiently than CAE (see Table 1). The flowchart of DCAE algorithm is shown in Fig 7. The details of DCAE are as follows. First, the input vector **x** passes through the dynamic convolution layer, the pooling layer and hidden layer to obtain an output vector **y**. This process is called encoding. The encoding formula is as follows:

$$t=g(\tilde{W}(x)⊗x+\tilde{b}(x))$$ (24)

y=subsampling(t) (25)

$$\tilde{W}(x)=\sum_{k=1}^{k}\pi_{k}(x){\tilde{w}}_{k}\;\tilde{b}(x)=\sum_{k=1}^{k}\pi_{k}(x){\tilde{b}}_{k}$$ (26)

$$s.t.\;0\leq \pi_{k}(x)\leq 1,\sum_{k=1}^{k}\pi_{k}(x)=1$$

where *Π*_*k*_ denotes the attention weight of the *K*-th linear function, ⨂ de-notes the convolution operation, **W** and **b** are the weight matrix and bias vector, g is the sigmoid activation function, $\tilde{W}$is the aggregation weight, and $\tilde{b}$is the aggregation bias.

Then, the input **y** passes through the deconvolution layer and the out-put layer to obtain the reconstructed vector **x’**. This process is called decoding. The formula for decoding is as follows:

$$x'=g({\tilde{W}}^{T}(x)⊗y+\tilde{b}(x))$$ (27)

During the training of each layer, we computed the loss function between the reconstruction vector **x’** and the input vector **x**, and optimized the value of the loss function to a threshold. An optimization process was performed at each layer.

The attention weights will vary according to **x** to obtain the optimal aggregation model. Therefore, the dynamic convolutional autoencoder can achieve better higher level representations than the ordinary autoencoder. The dynamic convolution consists of three parts, including attention weights, $\tilde{W}$ and $\tilde{b}$ in the optimal weights. In DCAE, the computational cost of the input feature ***H×W×C***_***in***_ is much smaller than that of ordinary convolution. The computational cost is as follows:

$$O(\pi (x))=HWC_{in}+C_{in}^{2}/4+C_{in}K/4$$ (28)

$$O(\tilde{W}x+\tilde{b})=HWC_{in}C_{out}D_{k}^{2}$$ (29)

where *O*(*•*) denotes computational cost, *D*_*k*_ denotes kernel size, *C*_*out*_ denotes the number of output channels. The computational cost of attention weights is much lower than directly calculating the optimal parameters. DCAE has better flexibility and lower computational cost than ordinary autoencoders.

In the experiment, we set the DCAE as a two-layer network with a learning rate of 0.001, using minimum mean squared error (MSE) as the loss function and gradient descent algorithm as the optimization method.

### Random forest classifier predicts associations

In the experiment, a random forest classifier used the extracted features to complete a classification task to discover potential circRNA-disease associations. The execution steps of the random forest classifier can be summarized as follows:

1. The classifier selects *N* samples using Bootstrap method. The selected *N* samples are used to train a decision tree.
2. The classifier randomly selects m features from the *M* features of the sample (*m* << *M*), and selects one feature from the *m* features as the split feature of the node using the information gain ratio. In the process of forming a decision tree, each node is split until it can no longer be split.
3. According to steps 1~2, a large number of decision trees are constructed to form a random forest.

The random forest classifier predicts scores for circRNA-disease associations. An association is considered a potential association if the prediction score is greater than a set threshold. The grid search algorithm was used to determine parameters in the classifier, and the number of decision trees was set to 100.

### Evaluation methods

The two commonly used methods (*k*-fold cross-validation and independent dataset testing) were used to evaluate the model performance. In the experiments, we recorded the true positive (TP), false negative (FN), true negative (TN) and false positive (FP) values. Five evaluation metrics were used to assess the model, namely area under curve (AUC), accuracy (ACC), sensitivity (Sen), F1-Score and Matthew correlation coefficient (MCC). These evaluation metrics are defined as follows:

$$\begin{array}{c} TPR=\frac{TP}{TP+FN}\\ FPR=\frac{FP}{FP+TN}\\ Acc=\frac{TP+TN}{TP+TN+FP+FN}\\ Sen=\frac{TP}{TP+FN}\\ MCC=\frac{TP\times TN-FP\times FN}{\sqrt{\left(TP+FP)(TP+FN)(TN+FP)(TN+FN\right)}} \end{array}$$ (30)

## Supporting information

S1 Table Comparison of running times of iCircDA-NEAE and iCircDA-NEAE. (DOCX)

S2 Table The 10-fold cross-validation experimental results on the circRNAdisease. (DOCX)

S3 Table The 10-fold cross-validation experimental results on the circ2Disease. (DOCX)
