# Supplementary material for: iCircDA-NEAE: Accelerated attribute network embedding and dynamic convolutional autoencoder for circRNA-disease associations prediction
Source: PLoS Comput Biol. 2023 Aug 31;19(8):e1011344. doi: 10.1371/journal.pcbi.1011344 (PMC10470932; doi:10.1371/journal.pcbi.1011344)
Supplement: S1 Table — (DOCX) [file pcbi.1011344.s001.docx]

**Supplementary Table 1.** Comparison of running times of iCircDA-NEAE and iCircDA-NEAE’

| Model | iCircDA-NEAE | iCircDA-NEAE’ |
| --- | --- | --- |
| Time | 63 min 27 s | 80 min 23 s |

Note: iCircDA-NEAE’: DCAE is replaced by CAE in iCircDA-NEAE.
