# Supplementary material for: iCircDA-NEAE: Accelerated attribute network embedding and dynamic convolutional autoencoder for circRNA-disease associations prediction
Source: PLoS Comput Biol. 2023 Aug 31;19(8):e1011344. doi: 10.1371/journal.pcbi.1011344 (PMC10470932; doi:10.1371/journal.pcbi.1011344)
Supplement: S2 Table — (DOCX) [file pcbi.1011344.s002.docx]

**Supplementary Table 2.** The 10-fold cross-validation experimental results on the circRNAdisease

| Test set | Acc | Sen | F1 | MCC | AUC |
| --- | --- | --- | --- | --- | --- |
| 1-fold  2-fold  3-fold  4-fold  5-fold  6-fold  7-fold  8-fold  9-fold  10-fold  Average | 0.8678  0.8601  0.8705  0.8679  0.8731  0.8826  0.8760  0.8814  0.8727  0.8833  0.8735 | 0.8349  0.8470  0.8393  0.8426  0.8565  0.8511  0.8154  0.7980  0.8689  0.8601  0.8413 | 0.8262  0.8220  0.8493  0.8107  0.8255  0.8019  0.8348  0.8585  0.8169  0.8281  0.8274 | 0.7016  0.6575  0.6654  0.6133  0.6841  0.6532  0.6686  0.6448  0.6989  0.6477  0.6635 | 0.9013  0.8816  0.8943  0.9065  0.8922  0.8873  0.8972  0.9038  0.8867  0.9111  0.8962 |
