# Supplementary material for: iCircDA-NEAE: Accelerated attribute network embedding and dynamic convolutional autoencoder for circRNA-disease associations prediction
Source: PLoS Comput Biol. 2023 Aug 31;19(8):e1011344. doi: 10.1371/journal.pcbi.1011344 (PMC10470932; doi:10.1371/journal.pcbi.1011344)
Supplement: S3 Table — (DOCX) [file pcbi.1011344.s003.docx]

**Supplementary Table 3.** The 10-fold cross-validation experimental results on the circ2Disease

| Test set | Acc | Sen | F1 | MCC | AUC |
| --- | --- | --- | --- | --- | --- |
| 1-fold  2-fold  3-fold  4-fold  5-fold  6-fold  7-fold  8-fold  9-fold  10-fold  Average | 0.8443  0.8409  0.8516  0.8483  0.8545  0.8629  0.8571  0.8620  0.8518  0.8635  0.8537 | 0.7487  0.7569  0.7483  0.7555  0.7694  0.7626  0.7258  0.7091  0.7782  0.7756  0.7530 | 0.7080  0.7017  0.7284  0.6910  0.7066  0.6835  0.7132  0.7357  0.6973  0.7089  0.7074 | 0.4735  0.4282  0.4367  0.3812  0.4533  0.4256  0.4379  0.4173  0.4694  0.4180  0.4341 | 0.8628  0.8421  0.8552  0.8674  0.8543  0.8480  0.8569  0.8642  0.8466  0.8775  0.8575 |
